# Onchocerciasis-associated epilepsy: another piece in the puzzle from the Mahenge mountains, southern Tanzania

**DOI:** 10.1186/s40249-019-0545-5

**Published:** 2019-05-24

**Authors:** Christoph Kaiser, Bruno P. Mmbando, Joseph N. Siewe Fodjo, Patrick Suykerbuyk, Mohamed Mnacho, Advocatus Kakorozya, William Matuja, Adam Hendy, Helena Greter, Williams H. Makunde, Robert Colebunders

**Affiliations:** 1 Pediatric Practice, Balzenbergstrasse 73, 76530 Baden-Baden, Germany; 2 National Institute for Medical Research, Tanga Research Centre, Tanga, Tanzania; 3 Global Health Institute, University of Antwerp, Antwerp, Belgium; 4 Muhimbili National Hospital, Dar es Salaam, Tanzania; 5 Enhance Tanzania Foundation, Dar es Salaam, Tanzania; 6 Muhimbili University of Health and allied Sciences, Dar es Salaam, Tanzania; 7 University of Texas Medical Branch,, Galveston, TX USA

## Abstract

In a study from the onchocerciasis-endemic area of Mahenge in southern Tanzania, Mmbando et al. [Inf Dis Poverty. 2018;7:64] demonstrate that in four selected villages the overall epilepsy prevalence was high, and significantly more elevated in the two villages of higher onchocerciasis endemicity compared to those of lower endemicity. This is replicating earlier findings from many other areas of tropical Africa. The authors are also providing data indicating that in the Mahenge focus, the prevalence of nodding syndrome may be related to that of onchocerciasis in the same way as epilepsy in general. The application of a clinical case definition for onchocerciasis-associated epilepsy (OAE) as used in the study of Mmbando et al. [Inf Dis Poverty. 2018;7:64] faces some difficulties; indeed, its precision in discerning cases of OAE from epilepsy due to other etiologies is not known, and it does not allow for a specific diagnosis in the individual patient. Because an operational surveillance tool for assessing the number of patients in the population could mean substantial advance for better estimating the burden of OAE, the proposed definition should be tried in different settings and its performance reviewed in the process.

**Electronic supplementary material:**

The online version of this article (10.1186/s40249-019-0545-5) contains supplementary material, which is available to authorized users.

## Multilingual abstracts

Please see Additional file 1 for translations of the abstract into five official working languages of the United States.

## Background

In an extensive and carefully performed study from the onchocerciasis-endemic area of Mahenge in southern Tanzania, Mmbando et al. [1] demonstrate that in four selected villages the overall epilepsy prevalence was high, and significantly more elevated in the two villages of higher onchocerciasis endemicity compared to those of lower endemicity. This is in line with previous evidence gathered from 91 communities examined within eight studies from seven countries in tropical Africa that epilepsy prevalence is increasing at a progressive rate with rising onchocerciasis prevalence [2]. The term of onchocerciasis-associated epilepsy (OAE) was suggested to designate the strong link between the two entities throughout West, Central and East Africa [2–4]. Interestingly, three points related to the clinical and epidemiological issues on onchocerciasis and epilepsy have been proposed by Dr. Christoph Kaiser from Germany, which need to be debated to clarify some terms for use in future studies. Therefore, Dr. Xiao-Nong Zhou, the Editor-in-Chief of *Infectious Diseases of Poverty*, decided to present both views of the debating sides, in order to improve the concepts related to the investigation of onchocerciasis and epilepsy in the developing world.

### How to measure the onchocerciasis transmission level?

Kaiser raised the question of how to adequately measure the onchocerciasis transmission level, due to following reasons: as a difference to most of the studies included in the review of Pion et al. [2] where onchocerciasis endemicity was assessed on the basis of dermal biopsies searching for live microfilaria of *Onchocerca volvulus* (mf), in the present study from Mahenge a serological tool measuring specific antibodies against *O. volvulus* antigen (Ov16 test) was used in a sample of village inhabitants at an age of more than 19 years and this was combined with palpation for onchocerciasis nodules [1]. Because the Ov16 test and nodule palpation have the disadvantage not to discern in the individual patient between active and inactive past infection, this was complemented with performing the Ov16 test also in the 6–10 year old children of the study villages [1]. Because of the long life expectancy and fecundity of the adult stages of *O. volvulus* of about 10 years [5], it must be assumed that those 38.4% of the children found with positive Ov16 tests in the two high-endemic villages had viable infections and onchocerciasis transmission in these locations is substantial whereas transmission is at a low level, though still ongoing, in the low-endemic villages, despite annual community-directed treatment with ivermectin (CDTI) since 1997 [1]. The procedure followed by Mmbando et al. is considered a useful and appropriate way for complementing or replacing time consuming skin snip surveys which require expertise in microscopy that is not readily available in many endemic areas.

Response of Mmbando et al.: The optimal way to determine the level of onchocerciasis transmission, as currently recommended by the World Health Organization, is to perform onchocerciasis antibody testing of children below the age of 10 years and/or to screen blackflies for the presence of infective L3 larvae or to screen them with the O150 PCR. Both the serological and an entomological study suggested high ongoing onchocerciasis transmission in rural villages in the Mahenge area.

### What is the relationship between epilepsy in general, nodding syndrome (NS), and onchocerciasis in the study area?

Within the overall group of patients with epilepsy, Mmbando et al. [1] identified a number of patients with a peculiar form of head nodding seizures, nodding syndrome (NS) [6]. Based on the definition of NS adopted at an international conference in Kampala in 2012, these patients were classified as probable cases of NS [7]. Interestingly, 12 patients with NS were found in the two villages highly endemic for onchocerciasis with a population of 2499 inhabitans, but only one patient in the two low-endemic villages with a population of 2618 inhabitants. A similar observation was made as early as 1994 in the Itwara onchocerciasis focus of western Uganda where eight patients with probable NS were found in three hyperendemic villages with a population of 1169 inhabitants, and four were found in ten villages of lower endemicity for onchocerciasis with 3574 inhabitants [8]. This is an indication that the positive correlation found between onchocerciasis and epilepsy prevalence in general [2] may also hold true for patients with NS.

Case-control data on the relationship between onchocerciasis and epilepsy in Mahenge are available from a survey conducted in 2005 [9, 10]. In this study, onchocerciasis infection was determined with skin biopsies of a group of patients with epilepsy and a control group of healthy caretakers of these patients by microscopy for mf, combined with an *O. volvulus* specific PCR test. The results of these examinations were analyzed for statistical significance in a systematic review [3] demonstrating an odds ratio of 3.77 (95% *CI*: 2.18–6.52) for mf microscopy alone [9] and an odds ratio of 4.36 (95% *CI*, 2.62–7.24) when mf microscopy was combined with PCR [10]. Studies examining onchocerciasis infection status of epilepsy patients and healthy controls in other endemic areas found similar associations of moderate, though reproducible, significance [3, 4]. There is evidence that this association is stronger with increasing infection intensity [3, 11–13]. In combination with the prevalence data now presented by Mmbando et al. [1], it can be concluded that the peculiar pattern of epilepsy in Mahenge fits with the general concept of OAE. The possible correlation between prevalence of NS, epilepsy in general and that of onchocerciasis should be systematically investigated in Mahenge and in other areas of high onchocerciasis endemicity.

Response of Mmbando et al.: We agree with Dr. Kaiser about the importance of more research on these issues. This should also include studies on other possible complications of onchocerciasis infection, in particular growth failure and neuro-endocrine disorders (Nakalanga syndrome) [14, 15].

### Is a case definition for OAE useful to improve differentiation between epilepsy due to cysticercosis and onchocerciasis?

As a shortcoming of the investigation of Mmbando et al. [1], it has to be mentioned that no sufficient efforts were made to assess the influence of possible alternative etiologies for the detected rates of epilepsy prevalence or incidence and their difference between study villages. This refers in particular to neurocysticercosis (NCC) which is known to be widespread in tropical Africa [16]. Although the authors report that in the study area pigs were raised, and this was mainly in the suburban villages (low-endemic for onchocerciasis), it remains unclear how this information was assessed and what would be the effect of this for the particular village. Besides NCC, in a number of patients epilepsy could as well be due to past cerebral malaria [17].

In an attempt to more precisely identify the proportion of those patients in whom epilepsy should be actually attributed to onchocerciasis, Mmbando et al. [1] used a definition for cases with OAE as “a previously healthy person who had developed epilepsy without an obvious cause between the ages of 3 and 18 years”. This definition was formulated by Colebunders et al. [18] for identification of those patients in an endemic area who should be considered to specifically represent cases of epilepsy due to *O. volvulus* infection. Based on anamnestic information, Mmbando et al. [1] found that in some of their patients an acute febrile illness or mental retardation had preceded the onset of epilepsy and conforming with their case definition of patients with OAE, concluded that in these cases seizures should be related to another obvious cause but not to onchocerciasis. Inversely, all remaining patients with seizure onset between 3 and 18 years were assumed to suffer from a form of epilepsy associated with, and implicitly induced by *O. volvulus* infection [1, 18]. This approach of a diagnosis by exclusion is neglecting the many forms of childhood epilepsies which are occurring worldwide through all ages [19] and cannot be easily identified on clinical grounds alone. Even more, it cannot be excluded that in the study area of Mahenge a substantial number of epilepsy cases could actually be affected by epilepsy due to NCC which does not leave out the age between 3 and 18 years [20–22].

Mmbando et al. [1] were applying the proposed case definition of OAE [1, 18] on those 27 patients with incidence of their first seizure during the 5 years preceding the survey and found its criteria fulfilled in 13 patients. Epilepsy incidence was slightly higher in villages of elevated onchocerciasis endemicity, but this difference was not significant either for all 27 patients or for the subgroup consistent with the proposed OAE definition [1]. In contrast with the results presented now from Mahenge [1], an earlier study on epilepsy incidence in 13 villages of the aforementioned Itwara focus [23] demonstrated a significantly elevated incidence in villages highly endemic of onchocerciasis (315 per 100 000 person-years) compared to villages of low endemicity (110 per 100 000 person-years; *P* < 0.01). Cysticercosis apparently did not play a major role in this area of western Uganda as indicated by a positive serology for *Taenia solium* found in only one of 53 examined patients with epilepsy [24]. To explain the discrepancy between the incidence data from Mahenge [1] and from western Uganda [23] it might be argued that in the study of Mmbando et al. [1] the difference of onchocerciasis endemicity between the compared villages was too weak, or the sample size was too small, to demonstrate an actually existing correlation. Alternatively, in Mahenge there might be one or several co-existing factors involved in epilepsy etiology or it could even be that there is no positive connection between onchocerciasis and epilepsy in the area. The data presented now by Mmbando et al. [1] allow no sufficient conclusion about this point.

As mentioned by Mmbando et al. [1], pig farming was found mainly in the two study villages of lower onchocerciasis endemicity and the comparatively even age distribution of epilepsy with a maximum at an age of 30 years found in these villages would be compatible with the age distribution in areas with NCC-associated epilepsy [22, 25, 26]. As a difference, the pattern observed in onchocerciasis-endemic areas is characterized by a peak in adolescents and young adults [24, 27] and this is similar to the age distribution observed by Mmbando et al. [1] in the villages of high onchocerciasis endemicity. However, because this was not assessed in sufficient detail by Mmbando et al. [1], the relevance of cysticercosis/ NCC in the study villages remains unclear, and it is impossible to make an estimate of how NCC could have interfered with the possible effect of onchocerciasis on epilepsy prevalence or incidence. The interactions between onchocerciasis, cysticercosis and epilepsy in Mahenge need to be further examined [28].

Response of Mmbando et al.: We confirm that the clinical case definition of OAE is unable to determine that seizures in an individual are caused by onchocerciasis. Indeed for clinical care, additional investigations such as neuroimaging are required to differentiate OAE from epilepsy due to neurocysticercosis.

This definition is however a very useful tool for epidemiological studies to identify onchocerciasis-endemic areas that should be prioritized for strengthening onchocerciasis elimination programmes and epilepsy care [29]. If in an onchocerciasis endemic area, a high prevalence and incidence of epilepsy is observed meeting the OAE criteria, this suggests that there is high *O. volvulus* transmission. Our study in Mahenge demonstrates this. It was already known since 1960 that there were many persons with epilepsy living in the Mahenge area [30], but no interventions were considered to prevent epilepsy in the region. Only recently, based on the results of our study, that the Tanzanian national neglected tropical disease control programme decided to implement semi-annual community directed treatment with ivermectin and is even considering using ground larviciding of rivers, as has been done in Uganda [29] to stop onchocerciasis and the incidence of OAE.

**Fig. 1:**
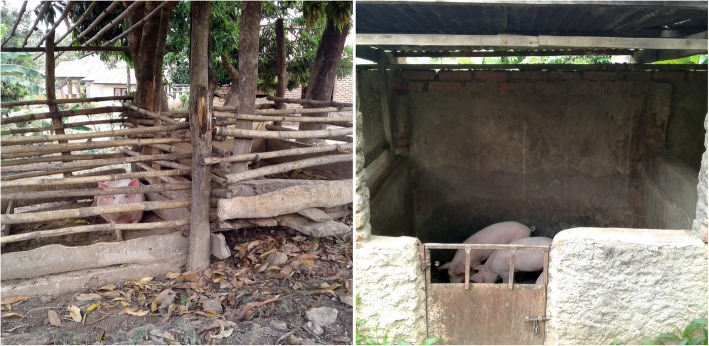
Small huts where pigs are kept in Mahenge

The higher prevalence of epilepsy in the rural villages in Mahenge with higher ongoing *O. volvulus* transmission, compared with the sub-urban villages, suggests that *O. volvulus* is the cause of the high epilepsy prevalence. We cannot exclude the presence of neurocysticercosis in the region; however, *Taenia solium* transmission may be low because pigs are generally kept and fed in small elevated huts preventing their contact with human stools (Fig. 1). Moreover, according to a socio-economic survey we performed in the two rural villages, only 6.2 and 9.1% of households in Mdindo and Msogezi respectively, were rearing pigs. Clinically, neurocysticercosis typically causes late-onset onset epilepsy [16] starting frequently after the adolescence period and is characterized mainly by focal seizures. In contrast, onchocerciasis-associated epilepsy is characterized by a majority of generalized tonic-clonic seizures without any obvious cause, that appear in previously healthy children between the ages of 3–18 years with a peak onset between the ages of 8–12 years as observed in Mahenge [1]. This peak of onset is not observed in non-onchocerciasis endemic regions in Africa. In the latter, most people develop epilepsy before the age of 5 years due to perinatal causes or epilepsy of genetic origin [4].

The value of the OAE clinical case definition is also illustrated by a recent epilepsy survey in Maridi county, an onchocerciasis-hyperendemic area in South Sudan where there are no pigs. In Maridi, an epilepsy prevalence of 4.4% [31] was observed with more than 85.2% of persons with epilepsy meeting the criteria of OAE [32]. This high percentage of epilepsy in Maridi, caused by OAE, is similar to the estimated high population attributable fraction of OAE (91.7, 95% *CI*: 56.7–98.4; *P* = 0.0021) in a longitudinal cohort study in Cameroon [13]. Another recent survey in two Nigerian villages with negligible onchocerciasis transmission made use of the proposed OAE definition to show that epilepsy among the native village residents was unlikely to be caused by onchocerciasis, as opposed to epilepsy observed among immigrant residents who had lived in onchocerciasis meso- or hyper-endemic areas before settling in the study villages [33].

## Conclusion

The study of Mmbando et al. [1] is inserting a new piece into the still fragmentary puzzle of OAE, in replicating results from many other areas of tropical Africa for the Mahenge mountains of southern Tanzania. For the first time, Mmbando et al. [1] are presenting data indicating that in onchocerciasis foci, the prevalence of NS may be related to that of onchocerciasis in the same way as epilepsy in general. The application of a clinical case definition as suggested by Colebunders et al. [18] should be further evaluated about its usefulness for identification of the proportion of epilepsy assumed to be induced by onchocerciasis in endemic areas (OAE). In particular, the capacity of this instrument to differentiate between OAE and NCC induced epilepsy or other forms of epilepsy needs to be tested. This could be helpful for a better quantitation of the total number of affected people, the disease burden attributable to OAE and of the effectiveness of onchocerciasis control on the health problem [34]. However, an operational case definition based on the firmly established epidemiological characteristics of OAE should be differentiated from a possible diagnosis of a specific form of epilepsy where infection with *O. volvulus* would be considered the proven cause for epileptic seizures in the individual patient. Research is needed to clarify the pathological mechanisms inducing seizures in the brain of patients with onchocerciasis. This would help to develop applicable tools allowing for a specific diagnosis of a clinical entity which then might be named “epilepsy induced by onchocercal brain disease” or “onchocerciasis induced epilepsy”. Although ample evidence has accumulated supporting the existence of such a condition, at present this is still to be considered a postulate.

Response of Mmbando et al.: Indeed, a recently performed retrospective cohort study in an onchocerciasis-endemic region in Cameroon showed that exposed children first acquire *O. volvulus* infection and later develop epilepsy, particularly those with a high microfilarial load, in a dose-response relationship [13]. It is important to determine the burden of epilepsy caused by *O. volvulus* and to map the major hotspots. As the highest prevalence of OAE is found in remote onchocerciasis-endemic areas where the healthcare infrastructure is very weak and where there are often problems of insecurity, this cannot be done with a complicated clinical definition that requires laboratory testing or imaging techniques. Therefore an easy-to-use clinical definition is needed to detect hotspots of ongoing *O. volvulus* transmission and to identify places where interventions are urgently needed to stop children from developing OAE.

## Additional file

Additional file 1: Multilingual abstracts in the five official working languages of the United Nations. (PDF 488 kb)
