# Supplementary material for: Onchocerciasis-associated epilepsy: another piece in the puzzle from the Mahenge mountains, southern Tanzania
Source: Infect Dis Poverty. 2019 May 24;8:35. doi: 10.1186/s40249-019-0545-5 (PMC6533739; doi:10.1186/s40249-019-0545-5)
Supplement: Supplementary file 1 — Multilingual abstracts in the five official working languages of the United Nations. (PDF 488 kb) [file 40249_2019_545_MOESM1_ESM.pdf]

Translation of the abstract into the five official working languages of the United Nations

تنزانيا جنوب ,مهانجي جبال في اللغز من أخر جزء :النهري العمى بداء المرتبط الصرع

كاكوروزيا، أدفوكاتوس مناتشو، محمد سويكربويك، باتريك فودجو، سيوي .إن جوزيف مباندو، .ب برونو كايزر، كريستوف
كولبوندرز وروبرت ماكوندي ويليامز ،هـ غريتر، هيلينا ،هندي آدم ماتوجا، ويليام

مختصرة نبذة

[Inf Dis Poverty. 2018;7:64] وآخرون إمباندو برهن ،تنزانيا بجنوب ماهينجي في النهري العمى داء توطن مناطق عن دراسة في أربع قرى، أن معدل انتشار الصرع في القريتين اللتين يستوطن فيهما العمى النهري بدرجة أعلى، كان مرتفعًا بشكل ملحوظ، وبالأخص مقارنةً بالقريتين اللتين يقل فيهما التوطن .وهذا يعد تكرارًا لنتائج سابقة من مناطق أخرى عديدة. كما أن بيانات الباحثين تقدم دليلًا على وجود علاقة محتملة بين معدل انتشار متلازمة الإيماء بالرأس وبين العمى النهري .الاستوائية أفريقيا في داء العمى النهري، فإن تعريف الصرع المرتبط بداء العمى النهري (OAE) وكما هو الحال بالنسبة للصرع، يفتقر إلى الدقة في سياق الواقع .وبشكل عام، عانى بعض من صعوبات؛ مثل الافتقار إلى الدقة في دراسة إمباندو وآخرون[Inf Dis Poverty. 2018;7:64] حالات بين التمييز لأن نظرًا وللمريض محدد شخيص تتيح لا والتي أخرى مسببات عن الناشئ الصرع وبين (OAE) الصرع المرتبط بداء حجم عبء الصرع لتقدير بالنسبة كبيرًا تقدمًا تعني قد السكان من المرضى عدد لتقييم التشغيلية المراقبة أداة .العملية هذه خلال أدائها ومراجعة مختلفة ظروف في المقترح التعريف تجربة في يتعين النهري، العمى بداء

Translated from English version into Arabic by Abdulrahman Al Sowaina, proofread by Amro Salah, through

盘尾丝虫病相关性癫痫：坦桑尼亚南部 Mahenge 山区的另一个难题

Christoph Kaiser, Bruno P. Mmbando, Joseph N. Siewe Fodjo, Patrick Suykerbuyk, Mohamed Mnacho, Advocatus Kakorozya, William Matuja, Adam Hendy, Helena Greter, Williams H. Makunde and Robert Colebunders

摘要：

Mmbando 等 [Inf Dis Poverty. 2018;7:64] 在坦桑尼亚南部 Mahenge 盘尾丝虫流行区的一项研究表明，4个被选定的村庄中癫痫的总体患病率很高，并且丝虫病流行程度较高的两个村庄的癫痫患病率明显高于流行程度较低的两个村庄。这与之前在热带非洲其他许多地区的发现相一致。作者的相关数据还表明，在 Mahenge 地区，同癫痫一样，点头综合征的患病率可能与盘尾丝虫病相关。在 Mmbando 等 [Inf Dis Poverty. 2018;7:64] 的研究中对盘尾丝虫病相关性癫痫（OAE）临床病例的定义应用存在一些困难。事实上，尚无法确切区分 OAE 和其他病因导致的癫痫，而且无法对个体患者进行特定诊断。因为开发一种可用于评估人群中患者数量的监测工具对于更好地评估 OAE 的卫生负担具有重要意义，所以应该在不同的环境中尝试提出不同的定义，并在整个过程中验证其效果。

Translated from English version into Chinese by Peng Song, edited by Pin Yang

Épilepsie associée à l’onchocercose : une autre pi èce du puzzle dans les monts Mahenge, au sud de la Tanzanie

Christoph Kaiser, Bruno P. Mmbando, Joseph N. Siewe Fodjo, Patrick Suykerbuyk, Mohamed Mnacho, Advocatus Kakorozya, William Matuja, Adam Hendy, Helena Greter, Williams H. Makunde et Robert Colebunders

Résumé

Dans une étude de la zone endémique de l'onchocercose de Mahenge, dans le sud de la Tanzanie, Mmbando et al. [Inf Dis Poverty. 2018;7:64] démontrent que la prévalence globale de l'épilepsie était élevée dans quatre villages choisis et significativement plus élevée dans les deux villages de plus forte endémicité de l'onchocercose que dans ceux d'endémicité plus faible. Cela reproduit des résultats antérieurs obtenus dans de nombreuses autres zones d'Afrique tropicale. Les auteurs présentent également des données qui indiquent que dans le point focal de Mahenge, la prévalence du syndrome du hochement de tête peut être liée à celle de l'onchocercose de la même manière que celle de l'épilepsie en général. L'application d'une définition de cas clinique pour l'épilepsie associée à l'onchocercose selon l'étude de Mmbando et al. [Inf Dis Poverty. 2018;7:64] se heurte à quelques difficultés. En effet, sa précision dans la discrimination de l'épilepsie associée à l'onchocercose et de l'épilepsie liée à d'autres causes n'est pas connue et elle ne permet pas un diagnostic spécifique de chaque patient individuel. Parce qu'un outil de surveillance opérationnelle pour l'évaluation du nombre de patients de la population constituerait un progrès considérable vers une meilleure estimation du fardeau de l'épilepsie associée à l'onchocercose, il serait utile de tester la définition proposée dans différents cadres et d'en évaluer les performances.

Translated from English version into French by Suzanne Assenat, proofread by Gwenaelle Le Jan-Moulart, through

TRANSLATORS WITHOUT BORDERS

Эпилепсия, вызванная онхоцеркозом: еще один ключ к разгадке в горах Махенге, южная Танзания

Кристоф Кайзер, Бруно П. Ммбандо, Джозеф Н. Сив Фоджо, Патрик Суйкербруйк, Мохамед Мначо, Адвокатус Какорозья, Уильям Матуйя, Адам Хэнди, Хелена Гретер, Уильямс Х. Макунде, и Роберт Коулбандерс

Аннотация

В отчете об областях с высокой заболеваемостью онхоцеркозом указан район обитания племени Махенге в южной Танзании. В отчете на примере показателей заболеваемости в четырех поселках, выбранных для проведения исследовательской работы, показано, что, в целом высок уровень заболеваемости эпилепсией. При этом заболеваемость онхоцеркозом проявляется значительно сильнее в двух из них, где возможность возникновения онхоцеркоза выше, чем в двух других изучаемых селениях [Ммбадо и др. «Инфекционные заболевания и бедность» 2018; 7:64]. Данные отчета отражают те выводы, которые были сделаны на основании изучения проблемы в других районах тропической Африки. Авторы исследования представляют также данные, указывающие на проблему племени Махенге: повышение уровня развития кивательного синдрома, который может рассматриваться как симптом онхоцеркоза, так и проявление эпилепсии. Описание клинического диагноза «эпилепсия, вызванная онхоцеркозом», предложенное в отчете [Ммбадо и др. «Инфекционные заболевания и бедность» 2018; 7:64], вызывает трудность в понимании данного медицинского понятия. Возникают проблемы при постановке диагноза в случае такого заболевания. Не установлено и его отличие от эпилепсии по этиологии, что не позволяет поставить

точный диагноз конкретному пациенту. Наблюдение за людьми, страдающими данным заболеванием, – важный этап в лечении пациентов. В свою очередь, для населения районов, подверженных онхоцеркозу, это может означать возможность оценки вреда здоровью от данного типа эпилепсии. Определение данного термина должно быть дано в дальнейшем в различных источниках.

Translated from English version into Russian by Elena Chavykina, proofread by Michael Orlov, through

TRANSLATORS
WITHOUT BORDERS

Epilepsia asociada a la oncocercosis: otra pieza del puzzle de las montañas Mahenge, sur de Tanzania

Christoph Kaiser, Bruno P. Mmbando, Joseph N. Siewe Fodjo, Patrick Suykerbuyk, Mohamed Mnacho, Advocatus Kakorozya, William Matuja, Adam Hendy, Helena Greter, Williams H. Makunde y Robert Colebunders

Resumen

En un estudio realizado por Mmbando et al. [Inf Dis Poverty. 2018;7:64] en la zona endémica de oncocercosis de Mahenge en el sur de Tanzania , se demuestra que en cuatro de las aldeas seleccionadas la prevalencia general de epilepsia fue alta y significativamente más elevada en las dos aldeas con mayor endemicidad de oncocercosis en comparación con las de menor endemicidad. Estos resultados replican las conclusiones anteriores de muchas otras áreas de África tropical. Asimismo, los autores proporcionan datos que indican que, en el foco de Mahenge, la prevalencia del síndrome del cabeceo podría estar relacionada con la de la oncocercosis de la misma manera que la epilepsia en general. La aplicación de una definición clínica del caso de la epilepsia asociada a la oncocercosis (OAE, por sus siglas en inglés), tal como se utilizó en el estudio de Mmbando et al. [Inf Dis Poverty. 2018;7:64], plantea algunas dificultades; de hecho, se desconoce su precisión a la hora de discernir los casos de OAE de los de epilepsia causada por otras etiologías y no permite un diagnóstico específico para cada paciente. Dado que una herramienta de vigilancia operativa para la evaluación del número de pacientes de una población podría suponer un avance substancial para estimar mejor la carga de la OAE, la definición propuesta debe probarse en diferentes contextos, del mismo modo que se debe revisar su funcionamiento a lo largo del proceso.

Translated from English version into Spanish by Celia Martinez, proofread by Mayra León, through

TRANSLATORS
WITHOUT BORDERS
